# Comparison and Evaluation of Annual NDVI Time Series in China Derived from the NOAA AVHRR LTDR and Terra MODIS MOD13C1 Products

**DOI:** 10.3390/s17061298

**Published:** 2017-06-06

**Authors:** Xiaoyi Guo, Hongyan Zhang, Zhengfang Wu, Jianjun Zhao, Zhengxiang Zhang

**Affiliations:** School of Geographical Sciences, Northeast Normal University, Changchun 130024, China; guoxy914@nenu.edu.cn (X.G.); zhaojj662@nenu.edu.cn (J.Z.); zhangzx040@nenu.edu.cn (Z.Z.)

**Keywords:** AVHRR LTDR V4, MODIS MOD13C1, annual NDVI, China, linear regression trends

## Abstract

Time series of Normalized Difference Vegetation Index (NDVI) derived from multiple satellite sensors are crucial data to study vegetation dynamics. The Land Long Term Data Record Version 4 (LTDR V4) NDVI dataset was recently released at a 0.05 × 0.05° spatial resolution and daily temporal resolution. In this study, annual NDVI time series that are composited by the LTDR V4 and Moderate Resolution Imaging Spectroradiometer (MODIS) NDVI datasets (MOD13C1) are compared and evaluated for the period from 2001 to 2014 in China. The spatial patterns of the NDVI generally match between the LTDR V4 and MOD13C1 datasets. The transitional zone between high and low NDVI values generally matches the boundary of semi-arid and sub-humid regions. A significant and high coefficient of determination is found between the two datasets according to a pixel-based correlation analysis. The spatially averaged NDVI of LTDR V4 is characterized by a much weaker positive regression slope relative to that of the spatially averaged NDVI of the MOD13C1 dataset because of changes in NOAA AVHRR sensors between 2005 and 2006. The measured NDVI values of LTDR V4 were always higher than that of MOD13C1 in western China due to the relatively lower atmospheric water vapor content in western China, and opposite observation appeared in eastern China. In total, 18.54% of the LTDR V4 NDVI pixels exhibit significant trends, whereas 35.79% of the MOD13C1 NDVI pixels show significant trends. Good agreement is observed between the significant trends of the two datasets in the Northeast Plain, Bohai Economic Rim, Loess Plateau, and Yangtze River Delta. By contrast, the datasets contrasted in northwestern desert regions and southern China. A trend analysis of the regression slope values according to the vegetation type shows good agreement between the LTDR V4 and MOD13C1 datasets. This study demonstrates the spatial and temporal consistencies and discrepancies between the AVHRR LTDR and MODIS MOD13C1 NDVI products in China, which could provide useful information for the choice of NDVI products in subsequent studies of vegetation dynamics.

## 1. Introduction

Vegetation, which plays a key role in the terrestrial ecosystem, maintains energy exchange and the global atmospheric, hydrologic, and carbon cycles in many ways [1]. Variations in vegetation constitute good indicators in ecological and environmental assessment from local to global scales [1,2,3,4]. In recent decades, monitoring changes and trends in vegetation have been improved by multiple remotely sensed datasets. Remote sensing technology provides unique insight into the dynamics of vegetation. Since the 1970s, diverse spectral indices have been extensively used to characterize the status of vegetation. Specifically, the Normalized Difference Vegetation Index (NDVI) exploits the difference in the spectral reflectance between the red and near-infrared (NIR) channels and is currently the most popular index. The NDVI is strongly related to the biophysical parameters of plants. Therefore, the NDVI has been successfully used to assess long-term vegetation dynamics and trends [1,5], detecting vegetation and climate interactions [6,7], and modeling the net primary production [8] and carbon balance of terrestrial ecosystems [9]. The observation of information by optical remote sensing is usually influenced by the scattering and absorption of the atmospheric composition [10,11] which causes the NDVI value to become smaller than its true value. The red and NIR spectral bands associated with NDVI are sensitive to the aerosol and water vapor content, respectively [12,13,14], particularly for sensors with broad spectral bands. Therefore, atmospheric correction is especially important to calculate NDVI values.

The National Oceanic and Atmospheric Administration (NOAA) Advanced Very High Resolution Radiometer (AVHRR) series sensors continuously monitor the status of the atmosphere, oceans, and land surface and have captured daily images of the earth from the 1980s to the present [15]. Resampled daily images are stored at a spatial resolution of 4.4 × 4.4 km^2^ in the Global Area Coverage (GAC) archive [15,16]. Various long-term and coarse-resolution NDVI time series products, such as Pathfinder AVHRR Land (PAL-I and II) [17,18], Fourier-Adjustment, Solar zenith angle corrected, Interpolated Reconstructed (FASIR) [19], Global Inventory Monitoring and Mapping Studies (GIMMS) [20] and its 3rd generation (3g) version, and Land Long Term Data Record (LTDR) [21], were developed based on the temporally and spatially comprehensive GAC images that were collected during the past three decades, although the series sensors were not initially designed for monitoring vegetation (but rather for meteorological studies) [15]. Many studies have investigated long-term vegetation changes by using various NOAA AVHRR NDVI products [22,23,24,25]. Different processing streams and correction schemes (e.g., atmospheric, bidirectional reflectance distribution function, and illumination) of the GAC images generated these NDVI products [26]. In this study, we focus on the LTDR NDVI product. The LTDR project (funded as part of the National Aeronautics and Space Administration (NASA) Earth Science Research, Education, and Applications Solutions Network) has generated daily top-of-atmosphere surface reflectance (AVH02C1), surface reflectance (AVH09C1) and vegetation index (AVH13C1) products from 1981 to the present. Currently, the GIMMS NDVI dataset is one of the most commonly used datasets for monitoring long-term vegetation dynamics among the various NOAA AVHRR NDVI products; however, this dataset may be replaced by the LTDR NDVI dataset in the near future [27] because the latter provides a finer spatial resolution, a broader geographical extent, a longer coverage period, and relatively perfect processing streams.

Since the late 1990s, newer sensors, such as the OrbView-2 Sea-viewing Wide Field-of-Sensor (SeaWiFS), Satellite Pour l’Observation de la Terre Vegetation (SPOT VGT), Terra/Aqua Moderate Resolution Imaging Spectroradiometer (MODIS), and Envisat Medium Resolution Imaging Spectrometer (MERIS), have also provided global NDVI time series products with various spatial and temporal resolutions. In recent years, studies of vegetation dynamics have strongly relied on the products from these newer sensors [3,7,28,29,30]. In particular, MODIS NDVI products with various spatial and temporal resolutions have been extensively applied for near-term (i.e., since 2000) vegetation monitoring. However, the short-term data records of MODIS NDVI products compared with those of NOAA AVHRR NDVI products represent a limitation for the long-term monitoring of vegetation trends. The merging of datasets that are produced by instruments with disparate specifications is highly challenging [20,31]. Thus, the NDVI products that are derived from NOAA AVHRR series sensors will be important to study the long-term dynamics of vegetation in the future.

Generally, NOAA AVHRR products, including PAL and GIMMS, have been integrated with Terra MODIS and SPOT VGT products to extend the period of coverage and improve the data quality of NDVI datasets [7,20,32,33]. The consistency of the four NOAA AVHRR NDVI products during 1982 and 1999 was intercompared and evaluated over the Iberian Peninsula and across the globe [15,26]. Additionally, previous studies focused exclusively on comparing the characteristics of the measured values, the seasonal and yearly trends, and the spatial patterns between AVHRR GIMMS and newer sensors NDVI products during an overlapping period at both the regional and global scales [4,27,32,34,35,36,37]. However, few studies focused on the comparison and evaluation of the spatial and temporal consistency of LTDR NDVI product with newer sensor products. Thus, the degrees of the spatial patterns and temporal trends of the consistency and discrepancies between the various NDVI products from AVHRR LTDR and MODIS MOD13C1 are important tasks that must be performed.

In this study, we focus on the annual NDVI which is the most generally used metric in long-term vegetation studies. The quality of the NDVI time series products from the AVHRR LTDR and MODIS MOD13C1 are assessed and compared in China by using overlapping datasets from 2001 to 2014. We aim to (1) understand the spatial patterns and the correlation of the NDVI values between the LTDR and MOD13C1 products and (2) reveal the trends of the LTDR and MOD13C1 NDVI products based on linear regression analysis during the 14-year period.

## 2. Data and Methods

### 2.1. AVHRR LTDR V4 Daily NDVI Product

The LTDR team has released four product versions since 2006 [21]. The last version (V4) products were made available (http://ltdr.nascom.nasa.gov) in 24 June 2014. The LTDR dataset was generated by GAC archive images from the NOAA AVHRR series sensors. The GAC time series images were reprocessed by using the preprocessing improvements that were identified in the PAL-II project [18]. The 6S radiative transfer model was applied to atmospherically correct the top of atmosphere AVHRR data to determine surface reflectance values for bands 1 and 2 including Rayleigh scattering, ozone absorption, water vapor and aerosol corrections [21]. A reanalysis dataset with a 0.5 × 0.5° resolution from the NOAA Center for Environmental Prediction (NCEP) was applied to water vapor corrections [38]. Aerosol climatology from internal NOAA was applied to correction for aerosol scattering [38]. One line element was used for the geometric correction of the LTDR V4 product [38]. Compared with previous versions of the LTDR dataset, V4 has improved the geolocation accuracy and is computed as the average of the available good data value observations in each grid cell [39]. The LTDR products from the NOAA AVHRR series sensors are available over a long period at a spatial resolution of 0.05 × 0.05°. Previous versions of the LTDR NDVI products have been used to map burned areas [16,40,41] and assess droughts [42].

The LTDR V4 NDVI product from January 2001 to December 2014, which was collected from AVHRR sensors aboard NOAA-16, 18, and 19, was used in the current analysis. Data records for some of the data days of LTDR V4 were removed from the archive because of geolocation errors or bad data quality. The unavailable data days are shown in Table 1. The percentage of available data records was over 90% of the days for each year. Each NDVI file consists of NDVI and quality assessment (QA) layers. The QA layer contains information regarding the overall cloud conditions and the validation of individual channel values for each pixel [21]. According to the documentation of the LTDR product [39], poor-quality NDVI pixels were removed based on the information in the QA layer; these pixels were influenced by cloud cover, cloud shadows or the invalidation of the channel 1 or 2 values. After removing the poor-quality pixels the mean percentage of spatial coverage for the daily record for China was approximately 25%. The high-quality pixels were primarily distributed in northern China, while southern China had fewer usable pixels because of cloud coverage. To reduce gaps or missing values from the long time series data, the Savitsky-Golay method was used to filter the NDVI product by using the TIMESAT program [43]. In addition, the maximum value composition (MVC) method was used to generate the annual NDVI dataset.

### 2.2. MODIS 16-Day Composite NDVI Product

Various MODIS products have been commonly used to monitor vegetation changes since 2000. In this study, MOD13C1 from the MODIS NDVI product (Collection 6), which covered a 14-year period from January 2001 to December 2014, was acquired from NASA’s Earth Observing System data gateway (http://reverb.echo.nasa.gov/reverb/). MOD13C1 is the Terra MODIS level 3 vegetation index product, which was calculated from the Terra MODIS level 2 surface reflectance product (MOD09 series). The atmospheric correction of MOD09 bands 1–7, which were based on the 6S radiative transfer model, was used to generate the surface reflectance [44]. The surface pressure, ozone and water vapor content were provided by NCEP for the atmospheric correction process of the MOD09 product. Additionally, the aerosols were derived from the MODIS data. The MOD13C1 product provides a global 16-day composite image at a spatial resolution of 0.05 × 0.05°.

The MOD13C1 product contains reliability and QA layers. The reliability layer contains a simplified ranking of the data and describes the overall pixel quality. The QA layer contains information regarding the quality, usefulness, aerosol quantity, and cloud conditions for each pixel. The pixels that are flagged as good (value equal to 1) and marginal (value equal to 2) quality in the reliability layer were retained for all the NDVI scenes. Pixels that were not flagged as good or marginal were removed by using the QA layer screening method as suggested by Arindam et al. [45] and Fensholt and Proud [46]. For each scene of MOD13C1 product, the mean percentage of spatial coverage was closed to 70% in China after removing the poor-quality pixels. The method of filling in the gaps values and annual NDVI composition were the same as for the LTDR V4 product preprocessing.

### 2.3. MODIS Land Cover Product

**Figure 1:**
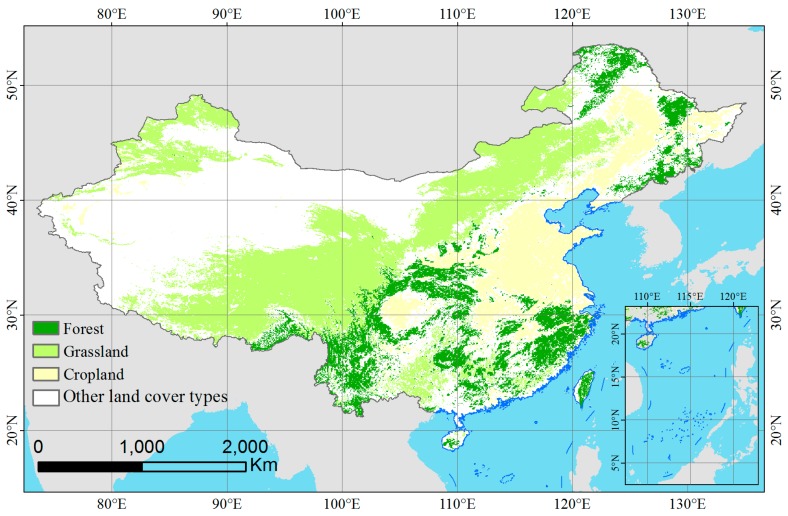
Spatial pattern of the major vegetation types in China based on the IGBP classification from the MCD12C1 product.

To compare the difference in the trends between the LDTR V4 and MOD13C1 NDVI products for various vegetation types, the MCD12C1 of the MODIS land cover product from 2001 to 2012 (all available years) was used to classify the land surface because the resolution (0.05 × 0.05°) of MCD12C1 matches the resolutions of the two studied NDVI products. Additionally, the annual MCD12C1 can provide accurate land cover for the following analysis. The MCD12C1 product was obtained from 2001 to 2012 and was processed from annual observation data from the Terra and Aqua MODIS sensors to depict the land cover types. This dataset consists of three different land cover classification schemes: the International Geosphere Biosphere Program (IGBP) classification, the University of Maryland classification, and the LAI/FPAR classification. The IGBP classification reflects the importance of natural vegetation among various land cover types. Therefore, we selected the IGBP classification scheme, which included 15 land cover classes (excluding deciduous needle-leaf forest and savannas) in China. Evergreen needle-leaf forest, evergreen broadleaf forest, deciduous broadleaf forest, and mixed forest were combined into one forest type. The few pixels that were classified as closed shrubland, open shrubland, and woody savannas in the MCD12C1 product of China were omitted from the following analysis. Therefore, the vegetation types were reclassified into three major types: forest, grassland, and cropland. We extracted the unchanged vegetation pixels from 2001 to 2012 to ensure the correction of the vegetation types during the study period. The resulting distribution of the major vegetation types in China is depicted in Figure 1.

### 2.4. MODIS Monthly Atmosphere Product

We obtained the MOD08_M3 product, which is a level 3 MODIS gridded atmosphere monthly joint product, with a 1 × 1° spatial resolution from January 2001 to December 2014 to reveal the effects of the atmospheric composition on the quality of the NDVI. This product contains atmospheric parameters that are related to the aerosol particle properties, total ozone burden, atmospheric water vapor, cloud optical and physical properties, and atmospheric stability indices. In this study, we extracted two datasets which were corrected aerosol optical depth (AOD) of land at 660 nm (red) spectral information and the total column precipitable water vapor (TCPWV). For the AOD dataset, some areas with high surface reflectance were characterized by null values because of MODIS dark target aerosol algorithms [47,48], such as deserts in northwestern China. The algorithm for water vapor values relies on the surface reflectance and transmittances of NIR bands (905 nm, 936 nm, and 940 nm) [49,50]. The mean of the daily mean value was selected as the monthly value for AOD and TCPWV. Then, we calculated the mean of the monthly value as the annual value from 2001 to 2014.

### 2.5. Statistical Analysis

Pearson’s correlation analysis was used to investigate the consistency between the composite annual NDVI dataset from the LTDR V4 and MOD13C1 products pixel by pixel from 2001 to 2014. The correlation coefficient (*r*) and slope were used to represent the strength and magnitude of the correspondence between the two datasets.

The temporal trends in the two NDVI datasets were determined by using linear regression analysis, with time as the independent variable and the annual NDVI value as the dependent variable for each pixel. This method has been widely used to investigate the dynamics of NDVI time series [1,46,51]. NDVI time series typically do not obey parametric assumptions such as normality and homoscedasticity [15]; therefore, a rank-based non-parametric statistical test, the Mann-Kendall (M-K) significance test, was used to assess the statistical significance of the temporal trends. The M-K significance test is less affected by missing values and uneven distributions than other test mohteds [52]. In this study, the NDVI time series were associated with a significant trend when the significance level was 95% (α = 0.05).

## 3. Results

### 3.1. Comparison of the Annual NDVI between the LTDR V4 and MOD13C1 Datasets

**Figure 2:**
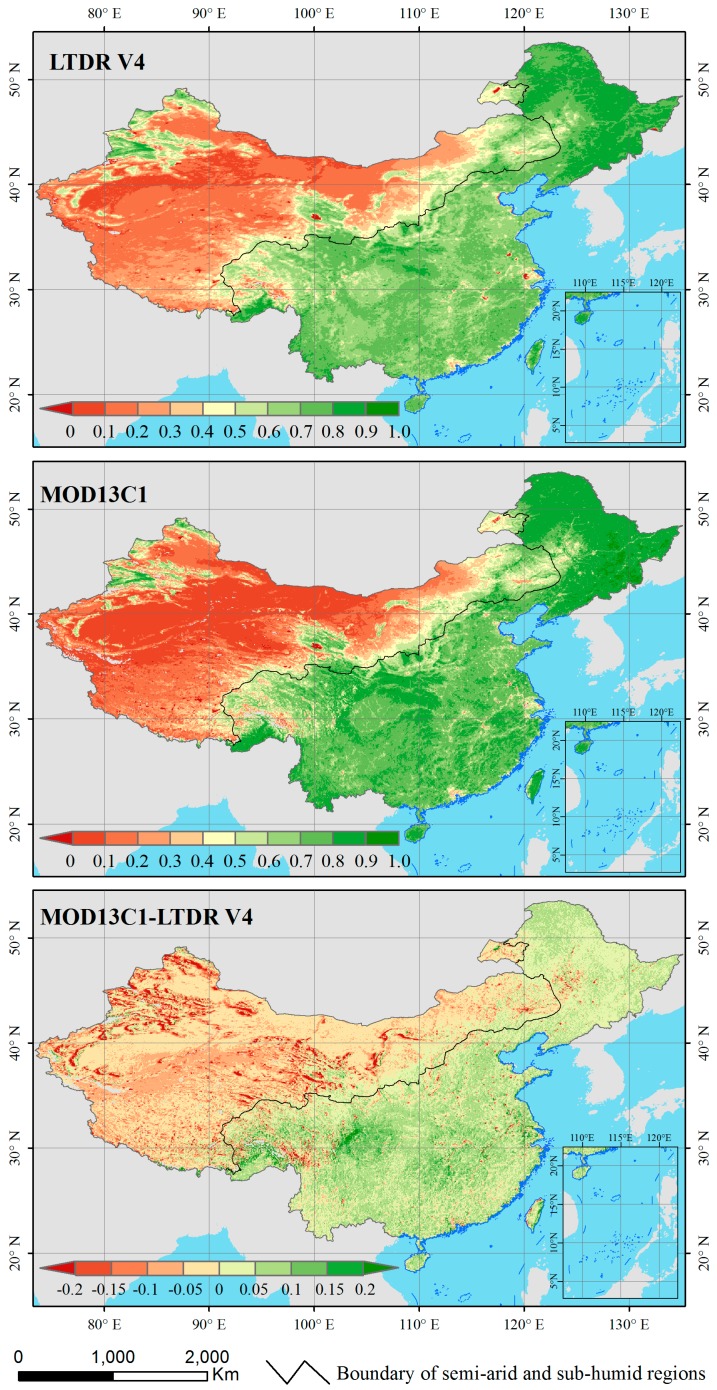
Spatial patterns of the average annual NDVI from 2001 to 2014 in China based on the LTDR V4 and MOD13C1 datasets and the difference average annual NDVI between MOD13C1 and LTDR V4.

Figure 2 illustrates the common spatial distributions of the NDVI and difference NDVI during 2001 and 2014 in China, revealing a general correspondence in the average annual NDVI between the LTDR V4 and MOD13C1 derived vegetation densities at the national scale. According to this figure, the transitional zone of NDVI values (high and low values) roughly matched the boundary of the semi-arid and sub-humid regions [53]. China can be divided into two regions according to this boundary, which are eastern and western China in this study. The annual NDVI values are relatively high for both products in most of eastern China. By contrast, the pixels that are characterized by low average annual NDVI values are primarily located in western China. However, some differences between both products can be observed, such as the magnitude of the NDVI values in western China. Obviously, the difference NDVI between MOD13C1 and LTDR V4 is higher in eastern China than that in western China.

**Figure 3:**
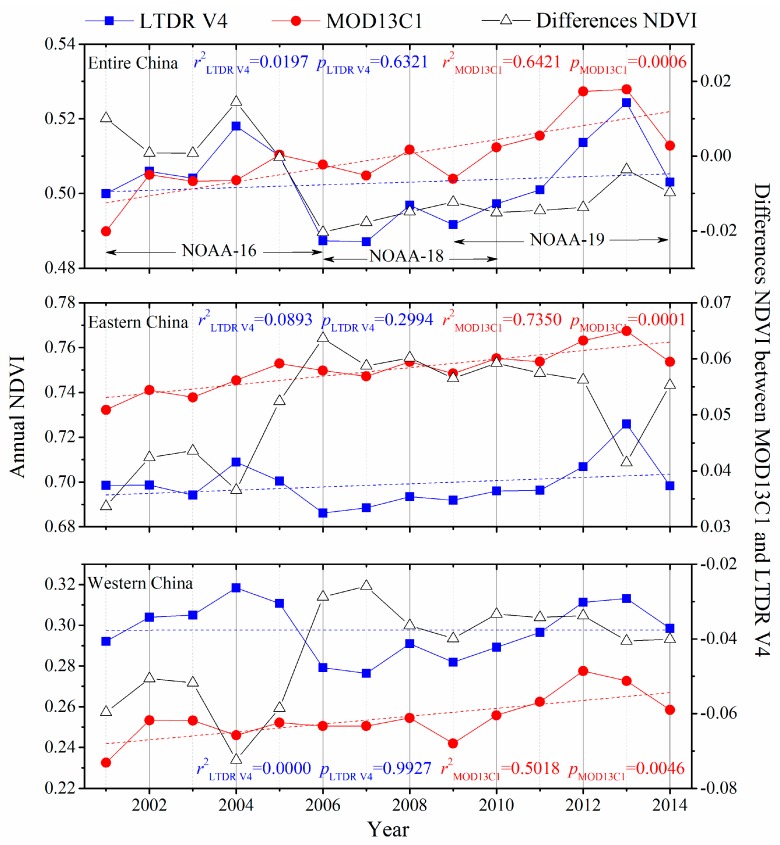
Variation in the inter-annual spatially averaged NDVI and difference NDVI based on the LTDR V4 and MOD13C datasets from 2001 to 2014 for all of China, eastern China, and western China.

The trajectories of the inter-annual variations in the spatially averaged NDVI values for all of China, eastern China, and western China from 2001 to 2014 are shown in Figure 3. During the study period, the spatially averaged annual NDVI from LTDR V4 displayed an insignificant trend. Conversely, the spatially averaged annual MOD13C1 NDVI significantly increased. The annual values of the LTDR V4 NDVI yielded a much lower *r*-value than those of the MOD13C1. A comparison of both trajectories revealed two distinct periods in the trajectory of the LTDR V4 dataset for all of China. From 2001 to 2005, the spatially averaged annual NDVI value from LTDR V4 was higher than that from MOD13C1, whereas the opposite pattern was observed from 2006 to 2014. Moreover, the spatially averaged annual NDVI value of LTDR V4 was obviously lower than that of MOD13C1 in eastern China for all of years of the study period, while the opposite result was observed in western China.

**Figure 4:**
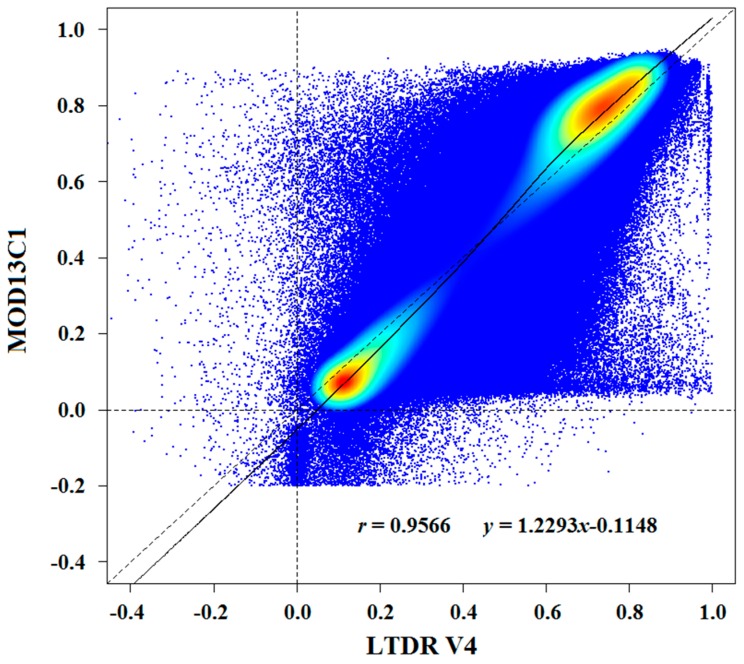
Density scatterplot of the annual NDVI values of the LTDR V4 dataset versus those of the MOD13C1 dataset for the 14-year period in China.

Pixel-by-pixel correlation analysis was performed on the annual NDVI of the LTDR V4 and MOD13C1 datasets from 2001 to 2014 over all of China. As shown in Figure 4, the LTDR V4 versus MOD13C1 NDVI values generally lay on the 1:1 line and collectively exhibited a dumbbell shape. A high correlation coefficient between the LTDR V4 and MOD13C1 NDVI values and a regression slope of approximately 1 were observed. However, some extremely large differences between individual observations were evident. The scatterplot shows that the regression line and the 1-to-1 line intersected at an NDVI value of approximately 0.5 for both datasets (Figure 4). In addition, the correlation of annual NDVI values between the two datasets was analyzed for each year from 2001 to 2014 (data not shown). The results indicated only slight year-to-year variability and a significant correlation coefficient (*r*) of approximately 0.95. Similarly, the regression slopes of the annual NDVI values showed high consistency between LTDR V4 and MOD13C1 in each year.

**Figure 5:**
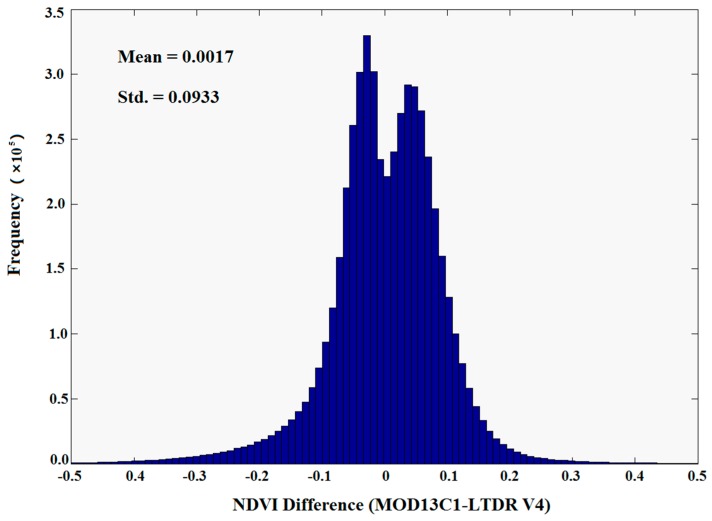
Frequency distribution of the differences between the MOD13C1 and LTDR V4 NDVI datasets from 2001 to 2014 in China.

The difference values of the NDVI are shown in Figure 5 and were calculated by subtracting the values of the LTDR V4 dataset from the corresponding values of the MOD13C1 dataset. Figure 5 shows that 80.59% of the pixel observations are within ±0.1 units.

### 3.2. Trends in the Annual NDVI in the LTDR V4 and MOD13C1 Datasets from 2001 to 2014

As described above, pixel-based linear regression analysis was performed on 14 years of LTDR V4 and MOD13C NDVI composite data from China. Table 2 shows that the positive trends of the pixels were generally more common than negative trends in both datasets from 2001 to 2014. The number of pixels with positive trends slightly exceeded the number with negative trends for the LTDR V4 NDVI dataset. The MOD13C1 NDVI dataset showed that over three quarters of China experienced positive trends and yielded a small number of pixels with negative trends. In total, 60.59% of the pixels displayed the same trends (either positive or negative trends) based on the LTDR V4 and MOD13C1 NDVI datasets, with more positive (45.44%) than negative trends. The LTDR V4 and MOD13C1 datasets displayed opposing trends for 39.41% of the pixels, primarily involving negative trends for LTDR V4 and positive trends for MOD13C1 (33.43%). By contrast, few pixels exhibited positive trends in LTDR V4 but negative trends in MOD13C1 (approximately 6%).

**Figure 6:**
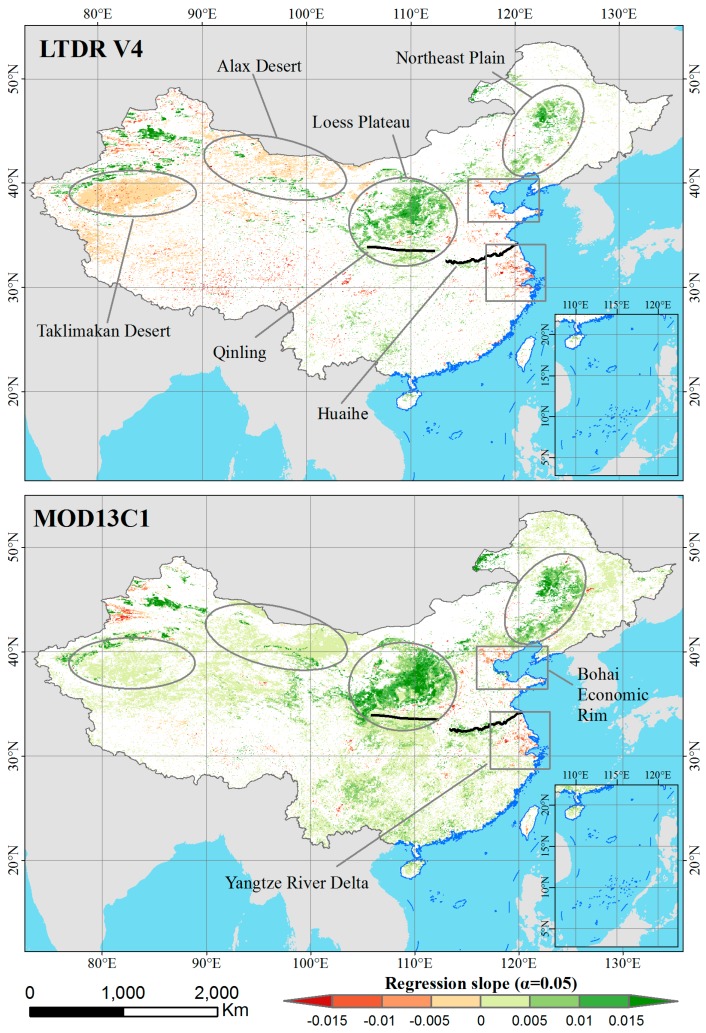
Linear trend regression slope values of the annual NDVI time series from 2001 to 2014 for the LTDR V4 and MOD13C1 datasets.

The M-K significance test was used to test the linear regression trends for the LTDR V4 and MOD13C13 NDVI datasets. Table 2 indicates that most areas in China showed insignificant trends from 2001 to 2014 for both datasets (α < 0.05). Figure 6 shows the spatial patterns of the significant trends for both datasets. A significant trend (increasing or decreasing) in the LTDR NDVI dataset was found for 18.54% of the study area. Among the pixels that showed increasing trends, the number that exhibited positive trends exceeded the number that exhibited negative trends by approximately 1% (Table 2). The MOD13C1 NDVI dataset included more pixels that exhibited significant trends than did the LTDR V4 dataset, with 35.79% of the study area showing significant trends. Approximately 94% of the MODIS pixels that showed significant trends also showed positive trends.

As shown in Figure 6, the spatial pattern of the significant NDVI trends showed obvious differences between the LTDR V4 and MOD13C1 datasets, particularly in western China. At the national scale, the pixels with significantly increasing trends were distributed in northern China based on the LTDR V4 dataset but were widely distributed throughout China based on the MOD13C1 dataset. At the regional scale, both datasets generally showed significantly increasing trends on the Northeast Plain and the Loess Plateau. Meanwhile, isolated regions with aggregations of pixels that showed significantly decreasing trends were found in eastern China in both datasets. Apparent differences existed in northwestern China, such as the Taklimakan Desert and the Alax Desert, in which significantly decreasing and increasing trends were observed in the LTDR V4 and MOD13C1 datasets, respectively. Additionally, significantly increasing trends were evident in most of southern China (southern Qinling-Huaihe Line) in the MOD13C1 dataset.

**Figure 7:**
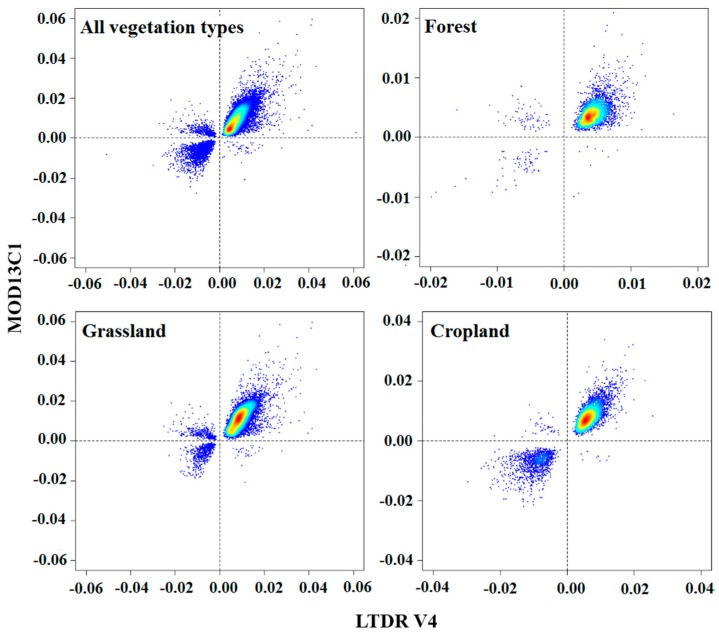
Density scatterplots of the linear trend regression slope values of LTDR V4 and MOD13C1 NDVI for pixels that corresponded to various vegetation types.

Figure 7 plots the significant linear regression slope values of the LTDR V4 and MOD13C1 datasets for vegetation in China. Generally, the significant linear regression slope values exhibited good agreement between both datasets for all vegetation pixels. A correlation existed between the NDVI regression slope values of LTDR V4 and MOD13C1 for all vegetation types, forest, grassland, and cropland. Almost all the vegetation pixels (over 97% of total) were located in the first and third quadrants. Vegetation pixels with positive slope values were more numerous than those with negative slope values. The significant linear regression slope values clearly differed among the vegetation types. For forest pixels, most of the slope values lay in quadrant 1. For grassland pixels, the slope values were more dispersed, with most in quadrants 1 and 3 and a smaller number in quadrant 2. For cropland pixels, an overall agreement existed between the datasets, with most of the slope values in quadrants 1 and 3. The magnitude of the regression slope value varied for the major vegetation types.

## 4. Discussion

The coarse spatial and high temporal resolutions of datasets such as NOAA AVHRR, Terra/Auqa MODIS and SPOT VGT enable us to understand the dynamics of the land surface at a broad scale. Dataset differences that are associated with the spectral range and preprocessing provide important insight into the reliability of the spatio-temporal characteristics of different datasets at various scales.

This study revealed a generally consistent spatial pattern of average annual NDVI in China between the LTDR V4 and MOD13C1 datasets (Figure 2). Previous analysis revealed similar spatial patterns in the NDVI between AVHRR GIMMS and MODIS products for northeastern Brazil [3] and northeastern China [33] during 2001 and 2006, respectively. The multi-annual mean and standard deviation of the NDVI in Europe displayed only slight differences between the AVHRR GIMMS 3g and MODIS NDVI products from 2001 to 2011 [34]. Considered together, the results of these studies indicate that the characteristics of the mean annual NDVI datasets (derived from AVHRR and MODIS) generally exhibited a relatively high agreement in the spatial distributions of various regions. Furthermore, phenological metrics [34] and gross primary productivity [54] from the AVHRR GIMMS and MODIS NDVI datasets have also been shown to exhibit similar spatial characteristics over the European continent and Southeast Asia, respectively. Therefore, we speculate that a comparison of secondary ecological parameters between AVHRR LTDR and MODIS might reveal similar spatial agreement.

The spatially averaged annual NDVI values indicated clear fluctuations and a significant increasing trend in the LTDR V4 and MOD13C1 datasets from 2001 to 2014 over China, respectively (Figure 3). The fluctuating trajectory of LTDR V4 might be explained by the transitions of the sensors during the 14-year period. In this study, the LTDR V4 dataset from 2001 to 2014 consisted of data that were obtained from three NOAA AVHRR sensors (NOAA-16, 18, and 19), whereas the MOD13C1 dataset was only covered by the Terra MODIS sensor. The spatial average of the LTDR V4 NDVI markedly decreased from 2005 to 2006, which coincided with the change from the NOAA-16 to the NOAA-18 sensor (Figure 3). Moreover, spatially averaged annual NDVI values from NOAA-16 AVHRR were larger than those from Terra MODIS, but the NDVI values from NOAA-18 and NOAA-19 exhibited opposing characteristics. Generally, the NDVI values from AVHRR sensors should be lower than those from MODIS sensor because of atmospheric effects. The NDVI values from NOAA-16 were probably anomaly. The data from the NOAA-16 sensor were possibly affected by noise and significant data gaps because of issues with the on-board scan motor, and a drift in the equator-crossing time caused a loss in the coverage of this sensor in the Northern Hemisphere [39].

The points in the scatterplot of the LTDR V4 versus MOD13C1 annual NDVI values from 2001 to 2014 were distributed near or along the 1-to-1 line (Figure 4). Our study found an *r*-value of 0.9566 between the LTDR V4 and MOD13C1 datasets for all the years. Atzberger et al. investigated the relationship between the NDVI values from the AVHRR GIMMS and MODIS datasets and found that the correlation coefficient was under 0.95 for not only all years combined (*r*^2^ = 0.88, *r* = 0.9380) but also each year (approximate *r*^2^ range: 0.85–0.90) from 2002 to 2011 [34]. The *r*-values indicate that the NDVI values between the LTDR V4 and MODIS datasets are more similar than those between the GIMMS and MODIS datasets, potentially because of differences in the spatial resolutions of various NDVI products. In the present study, the LTDR V4 and MOD13C1 datasets were compared at the same spatial resolution (0.05 × 0.05°) as that of the annual NDVI based on the MVC method. However, in reference [34], the compared NDVI products from AVHRR GIMMS and MODIS had spatial resolutions of 1/12° and 250 m, respectively, and an approximately bi-weekly temporal resolution.

**Figure 8:**
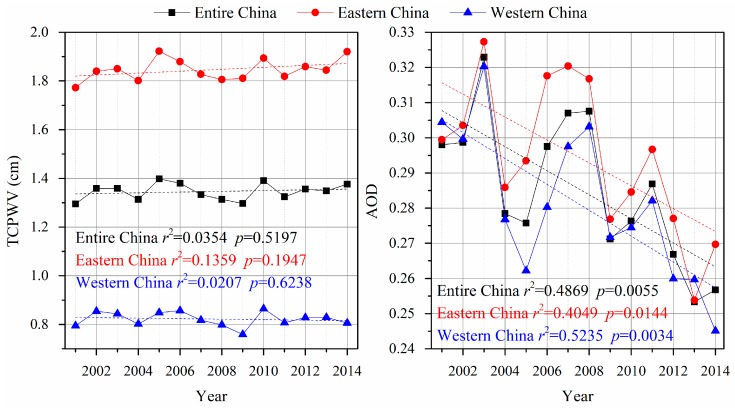
Variation in the inter-annual spatially averaged TCPWV and AOD from the MOD08_M3 product from 2001 to 2014 for all of China, eastern China and western China.

The slopes of the correlation analysis of the LTDR V4 NDVI values that were plotted against the MOD13C1 NDVI values and the AVHRR GIMMS NDVI values that were plotted against the MODIS NDVI values [34] were observed to be slightly greater than 1 during the study periods. These results are consistent with the fact that the values of the LTDR V4 and GIMMS pixels were frequently lower than those of the MODIS pixels. This result occurred because the NIR band exhibited differential sensitivities to the atmospheric water vapor effects between the AVHRR and MODIS sensors [55]. The NIR band of AVHRR (725 to 1000 nm) was much broader than that of MODIS (841 to 876 nm). The NIR bands of AVHRR sensors were significantly affected by water vapor absorption in the atmosphere, which caused the NDVI values to decrease in most of China. The narrower NIR band of the MODIS sensor avoided the atmospheric water vapor absorption range of the spectrum and was not affected by the water vapor contents. However, an interesting pattern was apparent in the density scatterplots of LTDR V4 versus MOD13C1 NDVI values (Figure 4): the lower range of NDVI values clustered below the 1-to-1 line, while the upper range of NDVI values clusters above the line. This pattern implies that the values of LTDR V4 were usually greater than those of MODIS over the lower range of NDVI levels, whereas the opposite was true for the upper range of NDVI values. This characteristic is certified by Figure 2 and Figure 3. The LTDR V4 NDVI values were greater than the MOD13C1 NDVI values in western China, which is characterized by the lower range of NDVI values. For the upper range of NDVI values in eastern China, the values of the LTDR V4 dataset were always smaller than those of the MOD13C1 dataset. Song et al. found that the NDVI values that were acquired by using the SPOT VGT sensors were typically lower than those that were measured with the AVHRR sensors in western China [32]. According to Figure 2, western China is located in arid and semiarid zones, which is characterized by a typical continental climate. Eastern China has a humid and sub-humid monsoon climate regime. The observed TCPWV values from the MOD08_M3 product in western China were clearly lower than those in eastern China according to Figure 8 (left). Consequently, the atmospheric water vapor content relatively weakly affected the NIR bands of AVHRR sensors in arid and semi-arid zones in western China. Huete et al. also verified that the AVHRR and MODIS NDVI values were nearly identical over arid and semi-arid sites in North America [56].

Because of the limitations the affected the period that was covered by prior AVHRR (i.e., GIMMS) and MODIS NDVI datasets, some previous studies used the integrated application of the two datasets to extend long-term time series [7,33,57]. This process enabled comparisons of the difference values of the NDVI time series between different datasets. Our results were also confirmed by analyzing the difference in the NDVI values from the AVHRR GIMMS and MODIS datasets at regional [7] and continental scales [34]. Song et al. analyzed the multi-year annual mean differences between the NDVI values that were acquired from the SPOT VGT and NOAA AVHRR sensors and obtained a mean difference value of 0.0111 [32]. These findings indicate reasonable pixel-by-pixel agreement between the various NDVI datasets.

More than half of China showed increasing trends in both the LTDR V4 and MOD13C1 datasets (Table 2). Several previous studies reported that NDVI trends corresponded to climate variations in China in recent decades [58,59,60,61]. In particular, rising temperatures were shown to strongly promote increasing NDVI values at the national scale. Since the 1980s, the annual mean surface temperature in China during the growing season has increased by 1.8 °C, with a warming rate of 0.062 °C per year [61]. Meanwhile, the atmosphere has been well known to introduce large variations in the NDVI values, independent of the surface vegetation conditions [62,63]. The detailed temporal changes in the TCPWV and AOD from 2001 to 2014 over China are presented in Figure 8. The annual spatially averaged TCPWV and AOD showed an insignificant trend and a significantly decreasing trend, respectively. These effects indicated that the observed NDVI values tended to increase over the study period. Direct human activities also played critical roles in some regions where the NDVI values showed consistent significant trends in both datasets. For example, the NDVI rapidly increased on the Loess Plateau because of rising temperatures and afforestation [61]. The Grain for Green project has considerably contributed to the nearly two-fold increase in vegetation cover from 1999 and 2013 based on the analyzed satellite imagery [64]. Gao et al. showed that the afforestation area totaled 95,804 km^2^ in Shanxi and Shaanxi Province, primarily on the plateau [65]. Agricultural practices may have been responsible for the significantly positive trends in the NDVI values on the Northeast Plain, which is an important commodity grain base in China. Irrigation and fertilization have promoted crop growth in these regions. The isolated pixels with significantly decreasing NDVI trends involved areas of rapid urban expansion in regions in eastern coastal China; these regions included the Yangtze River Delta and the Bohai Economic Rim (Figure 6), which are undergoing the largest societal and economic changes in the country.

In southern China, obvious discrepancies in the NDVI trends between the LTDR V4 and MOD13C1 datasets were evident (Figure 6), with many more pixels showing significantly positive trends in MOD13C1 than in LTDR V4. Southern China is located in a humid zone. On the one hand, these discrepancies could have been caused by differences in cloud-masking processes between datasets. Fensholt et al. reported that cloud masks of the NOAA AVHRR NDVI original products were deficient in areas of high humidity [27]. Applying insufficient cloud-masking procedures to the 14-year time series of satellite products could lower the resulting NDVI values and indirectly alter the NDVI trends [27]. On the other hand, a number of poor-quality pixels were present in southern China, which caused fewer pixels to be applied when for calculating the annual NDVI values.

Reference [27] reported that the trend of the NOAA AVHRR GIMMS average NDVI product during 2000 and 2007 was characterized by negative values in the desert area of the northernmost part of the Sahelian region, whereas the Terra MODIS product was characterized by positive values. Similar findings (Figure 6) were found in our study, with contrasting NDVI trends between the LTDR V4 and MOD13C1 datasets in a portion of the western desert region of China. The NDVI time series from LTDR V4 showed declines in these regions from 2001 to 2014, whereas that of MOD13C1 showed increases. The NDVI values of the AVHRR and MODIS products appear to exhibit opposing trends for desert pixels on various continents, although different studies have used different types of trend tests. In this study, the clear trends in desert areas were primarily caused by the long-term insufficient calibration of the orbital drift in the data from the NOAA AVHRR sensors. Therefore, the slopes of the linear regression analysis for the NDVI products do not reliably reflect the vegetation dynamics in desert areas but rather the magnitude of the calibration problems of the datasets from the AVHRR and MODIS sensors [27].

The plots of the NDVI regression slope from the two datasets that were produced according to the vegetation type (Figure 7) clearly showed agreement between the trends of the LTDR V4 and MOD13C1 NDVI, indicating that the overall agreement for all the vegetation pixels could be extended to various vegetation types. Therefore, the temporal trends from both datasets agreed well and could be used to detect long-term trends in the NDVI in most Chinese forest, grassland and cropland. The trend estimates of vegetation from previous studies generally agreed with our results. For most of the vegetation pixels, the trends from the LTDR V4 and MCD13C1 datasets were positive. The growing season NDVI increased in all ecosystems (vegetation types) except desert during 1982 and 2010 in China [59]. For forest, grassland, and cropland, the mean regression slope of the NDVI ranged from 0.0033 to 0.0132 and from 0.0060 to 0.0092 for the MOD13A3 and SPOT VGT datasets during 2001 and 2011, respectively [37]. A relatively low agreement in the regression slope values was observed in the grassland. A few pixels in quadrant 2 indicated grassland pixels with decreasing trends in LTDR V4 and increasing trends in MOD13C1. Although, the temperature was the primary factor for the dynamics of the NDVI at a broad scale. Vegetation growth was also relatively strongly affected by precipitation in the grassland [59,61]. The increase in precipitation implies an increase in the atmospheric water vapor content at a local scale [66]. As described above, the NIR bands of the AVHRR series sensors were more sensitive than that of the MODIS sensor in terms of the atmospheric water vapor content. For certain grassland pixels, the atmospheric water vapor content caused the degradation of the NDVI values from LTDR V4 dataset at a local scale but barely influenced the NDVI values from the MOD13C1 dataset.

## 5. Conclusions

The LTDR NDVI product, which spans from 1981 to the present, may be a potentially useful NOAA AVHRR-based dataset to study long-term vegetation dynamics in the future. The composited annual time series NDVI of LTDR V4 was compared to that of MOD13C1 for an overlapping period of 14 years in a heterogeneous study area in China.

The spatial patterns of the average annual NDVI from LTDR V4 generally matched those from MOD13C1 at a national scale. The correlation analysis between these datasets had a high correlation coefficient based on pixel analysis. The spatially averaged NDVI time series of the MOD13C1 dataset showed significant increases over the 14-year period for all of China, eastern China and western China; however, insignificant increases were observed in the corresponding LTDR V4 dataset. The observed NDVI values of LTDR V4 were lower than those of MOD13C1 in eastern China because of the effect of the atmospheric water vapor content on the NIR bands of AVHRR series sensors; the opposite trend was observed in western China. A shift in the spatially averaged annual NDVI was observed for the LTDR V4 dataset from 2005 to 2006 because of a change to the NOAA AVHRR sensor. A significant trend in the NDVI was found in 18.54% of the LTDR V4 pixels and 35.79% of the MOD13C1 pixels. The spatial distributions of the significantly positive trends were similar between the two datasets for the Loess Plateau and Northeast Plain, and those of the negative trends matched well in eastern China. Larger discrepancies in the trends between datasets were identified in China’s desert area and in southern China. Furthermore, this study suggests that LTDR and MOD13C1 NDVI are highly consistent in detecting linear regression trends in various vegetation types.

Here, we highlighted the consistencies and differences between the two datasets rather than quantitatively determining whether one dataset was superior. Thus, this study provides a baseline for future studies, which should focus on the agreement among estimations of the phenology, productivity, FPAR, and vegetation fraction cover from the AVHRR LTDR and MODIS NDVI products.

## Figures and Tables

**Table 1:** Data days are removed from LTDR V4 during the study period.

Year	Julian Day
2001	23
2002	11, 226, 289
2003	71, 237, 262, 263, 265
2004	46, 58, 81, 82, 85, 89, 90, 91, 92, 93, 94, 95, 96, 99, 100, 101, 118, 124, 126, 129, 134, 136, 140, 141, 142, 188, 196, 197, 199, 210
2005	38, 72, 96, 108, 113, 184, 187, 202, 214, 222, 235, 244, 269, 272, 280, 284, 320
2006	40, 41, 70, 71, 80, 103, 159, 160, 161, 162, 169, 170, 188, 189, 198, 199, 208, 209, 218, 219, 228, 229, 233, 277, 279, 297, 307
2007	1, 20, 30, 33, 59, 69, 108, 118, 134, 166, 167, 186, 195, 196, 205, 206, 225, 244, 264, 283, 293, 311, 312, 317, 341
2008	63, 73, 121, 122, 131, 149, 160, 169, 179, 198, 199, 207, 208, 209, 210, 211, 226, 227, 246, 255, 256, 257, 258, 259, 265, 285, 332
2009	4, 5, 24, 72, 81, 91, 92, 100, 110, 111, 119, 129, 138, 139, 157, 224, 262, 272
2010	5, 15, 24, 44, 63, 83, 142, 161, 162, 170, 171, 190, 191, 199, 200, 210, 219, 220, 229, 230, 239, 249, 250, 336, 337
2011	29, 30, 193, 194, 213
2012	-
2013	-
2014	-

**Table 2:** Percentage of pixels in China that exhibited positive and negative trends in the LTDR V4 and MOD13C1 NDVI datasets.

	LTDR V4		MOD13C1	
	All Pixels	Significant Pixels	All Pixels	Significant Pixels
Positive trends	50.81%	9.80%	78.82%	33.56%
Negative trends	49.19%	8.74%	21.18%	2.23%
